# The α-(1,3)-glucan synthase gene *agsE* impacts the secretome of *Aspergillus niger*

**DOI:** 10.1007/s10482-023-01853-w

**Published:** 2023-06-14

**Authors:** Jun Lyu, Costanza Torchia, Harm Post, Juan P. Moran Torres, A. F. Maarten Altelaar, Hans de Cock, Han A. B. Wösten

**Affiliations:** 1 Microbiology, Utrecht University, Padualaan 8, 3584 CH Utrecht, The Netherlands; 2 Biomolecular Mass Spectrometry and Proteomics, Bijvoet Center for Biomolecular Research and Utrecht Institute for Pharmaceutical Sciences, Utrecht University, Padualaan 8, 3584 CH Utrecht, The Netherlands

**Keywords:** Fungus, *Aspergillus niger*, Secretome, Colony morphology, Cell wall, α-(1,3)-glucan

## Abstract

*Aspergillus niger* is widely used as a cell factory for the industrial production of enzymes. Previously, it was shown that deletion of α-1–3 glucan synthase genes results in smaller micro-colonies in liquid cultures of *Aspergillus nidulans*. Also, it has been shown that small wild-type *Aspergillus niger* micro-colonies secrete more protein than large mirco-colonies. We here assessed whether deletion of the *agsC* or *agsE* α-1–3 glucan synthase genes results in smaller *A. niger* micro-colonies and whether this is accompanied by a change in protein secretion. Biomass formation was not affected in the deletion strains but pH of the culture medium had changed from 5.2 in the case of the wild-type to 4.6 and 6.4 for Δ*agsC* and Δ*agsE,* respectively. The diameter of the Δ*agsC* micro-colonies was not affected in liquid cultures. In contrast, diameter of the Δ*agsE* micro-colonies was reduced from 3304 ± 338 µm to 1229 ± 113 µm. Moreover, the Δ*agsE* secretome was affected with 54 and 36 unique proteins with a predicted signal peptide in the culture medium of MA234.1 and the Δ*agsE,* respectively. Results show that these strains have complementary cellulase activity and thus may have complementary activity on plant biomass degradation. Together, α-1–3 glucan synthesis (in)directly impacts protein secretion in *A. niger*.

**Supplementary Information:**

The online version contains supplementary material available at 10.1007/s10482-023-01853-w.

## Introduction

Aspergilli like *Aspergillus niger, Aspergillus oryzae, Aspergillus awamori, Aspergillus sojae,* and *Aspergillus terreus* are widely exploited as cell factories for the production of enzymes and metabolites (Meyer et al. 2011; Wösten et al. 2013; 2019). To this end, aspergilli are grown in bioreactors. In such cultures, aspergilli can grow as micro-colonies, also known as pellets, as a dispersed mycelium or in an intermediate state of pelleted and dispersed growth called clumps.

Pellet formation in bioreactors is the result of primary and secondary aggregation. These stages involve the interaction of non-germinated and germinated conidia, respectively (Grimm et al. 2004). Primary aggregation results from the hydrophobic nature of conidia due to the presence of hydrophobin and melanin layers at the outer surface of the spores. Inactivation of the *A. nidulans* hydrophobin genes *dewA* and *rodA* reduces aggregation of conidia and, as a consequence, results in smaller micro-colonies (Dynesen & Nielsen 2003). Similarly, inactivation of the melanin synthesis gene *olvA* in *A. niger* results in reduced primary aggregation and smaller micro-colonies (van Veluw et al. 2013). Medium composition, agitation, and pH can also affect primary aggregation of *Aspergillus* conidia (Carlsen et al. 1996; Metz & Kossen 1977) by impacting the hydrophobic interactions between the spores in the culture medium. Secondary aggregation results from the presence of α-(1,3)-glucan at the surface of germinating conidia of aspergilli (Fontaine et al. 2010; He et al. 2014; Yoshimi et al. 2013). Synthesis of α-(1,3)-glucan in *A. nidulans* is mediated by the α-(1,3)-glucan synthases AgsA and AgsB and the α-amylases AmyD and AmyG*.* Inactivation of the main α-(1,3)-glucan synthase gene *agsB* has no obvious phenotype on agar medium but reduces aggregation of germlings and, as a consequence, results in smaller micro-colonies in liquid shaken cultures (He et al. 2014; Yoshimi et al. 2013). Inactivation of the *A. nidulans* α-amylase gene *amyG*, but not *amyD*, also reduces secondary aggregation (He et al. 2014). This effect has been explained by assuming that this gene is involved in the synthesis of the primer structure of α-glucan (Marion et al. 2006).

A relation between morphology of the mycelium and yield of secreted enzymes or metabolites has been described. Initially, these results were based on studies where the morphology of the mycelium was changed by varying the culture conditions (Krijgsheld et al. 2013). Therefore, changes in yield and secretome composition could also have been the result of the changed culture conditions. Recently, two studies established a direct relation between mycelium morphology and productivity. By sorting large and small micro-colonies from the same liquid shaken culture it was shown that expression of genes of secreted proteins mainly occurs in a peripheral shell of the pellet (Tegelaar et al. 2020). Results indicated that pellets with a radius ≤ of the width of the expression shell would release more secreted protein. In a study of Lyu et al. (2023), mycelium morphology was controlled by different periods of pre-growth in alginate beads. Small micro-colonies secreted a higher amount and diversity of proteins in the culture medium when compared to large micro-colonies. Although the large micro-colonies were less productive, they did release their own unique set of proteins. In fact, cellulases released by large and small micro-colonies showed synergistic activity.

Here, the effect of deletion of *A. niger* α-(1,3)-glucan synthase genes on biomass formation, morphology, pH of the culture medium and the secretome was assessed. To this end, *agsC* and *agsE* were deleted since these two out of five α-(1,3)-glucan synthase genes are highly expressed in the first hours of growth in a liquid medium with xylose (Yuan et al. 2008). Inactivation of *agsC* only resulted in a lower pH of the culture medium, whereas deletion of *agsE* impacted micro-colony morphology as well as pH and the protein profiles in the culture medium. Together, data show that cell wall synthesis (in)directly impacts secretion of proteins in the culture medium.

## Material and methods

### Strains and culture conditions

*Aspergillus niger* strains (Table 1) were grown for 3 days at 30 °C on PDA plates. Conidia were harvested using a cotton swab and suspended in minimal medium (MM) (Wang et al. 2015) or transformation medium (TM; MM with 0.5% yeast extract and 0.2% casamino acids) with 25 mM xylose (MM-X and TM-X, respectively). Hyphae were removed from the spore suspension by filtering through a syringe with cotton. Conidia were counted using a haemocytometer, after which 2 × 10^7^ spores were introduced in 50 ml TM-X in 250 ml Erlenmeyer flasks either or not in the presence of doxycycline. After 16 h of growth, the mycelium was transferred to 100 ml MM-X and growth was prolonged for 32 h. Biomass and culture medium were separated by using a 40 μm cell strainer (Corning, 352,340, New York, United States).

**Table 1:** *A. niger* strains used in this study

Strain	Genotype	Description	Reference
MA234.1	ΔkusA:DR-amdS-DR, pyrG	3.8 kb *XbaI* pyrG gene replacement in MA169.4	Park et al. 2016
LZ04	Δ*agsC*	Deletion of *agsC* in MA234.1	This study
LZ05	Δ*agsE*	Deletion of *agsE* in MA234.1	THIS study
LZ06	*Tet-on:agsE*	Inducible *agsE* in MA234.1	this study

### Gene editing in A. niger

**Fig. 1:**
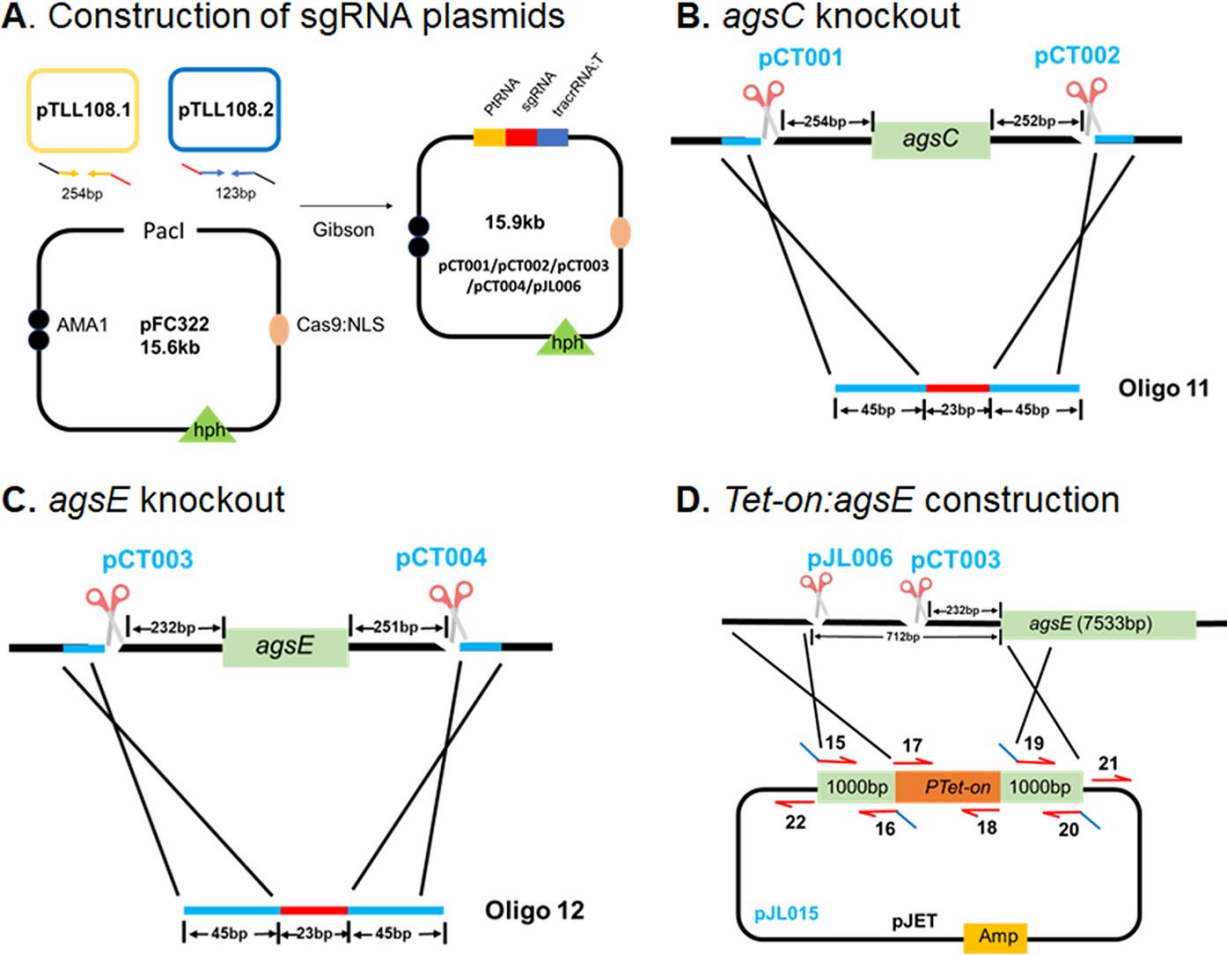
Construction of sgRNA plasmids (**A**) to delete *agsC* (**B**) and *agsE* (**C**) and to replace the *agsE* promoter for that of the inducible Tet-on system (**D**)

A CRISPR-Cas system (Nødvig et al. 2018) was used to inactivate *agsC* (ATCC64974_40950, AspGD) and *agsE* (ATCC64974_10510, AspGD) of *A. niger* and to replace the promoter of the latter gene for the inducible Tet-on cassette. For gene inactivation, *agsC* sgRNA’s sg01 and sg02 and *agsE* sgRNA’s sg03 and sg04 (Table 2) were designed using CHOPCHOP (https://chopchop.cbu.uib.no) to produce a double cut 254 and 232 bp before the start codon of *agsC* and *agsE* and 252 and 251 bp after the stop codon of these genes, respectively (Fig. 1BC). The sgRNA’s were placed under control of the RNA pol III promoter in plasmid pFC332 (Table 3) that also contains *cas9* and a hygromycin resistance cassette (Nødvig et al. 2018; van Leeuwe et al. 2019)(Fig. 1A). The sgRNA expression cassettes were amplified in two parts using plasmids pTLL108.1 and pTLL109.2 (van Leeuwe et al. 2019) (Table 3) as templates for the 5’ and 3’ fragments consisting of the PacI::Pro-promoter::sgRNA and sgRNA::Terminator::PacI, respectively. Primer pairs 1/4 and 2/3 (Table 4) were used to amplify the 5’ and 3’ fragments followed by their assembly into a PacI-linearized and dephosphorylated pFC332 vector using a Gibson reaction (Seekles et al. 2021) resulting in the *agsC* 5’ cutting sgRNA plasmid pCT001 (Table 3). Similarly, primer pairs 1/6 and 2/5 (Table 4) were used to produce the *agsC* 3’ cutting sgRNA plasmid pCT002 (Table 3). Primer pairs 1/8 and 2/7 and 1/10 and 2/9 (Table 4) resulted in the *asgE* 5’ and 3’ cutting sgRNA plasmids pCT003 and pCT004, respectively (Table 3).

**Table 2:** sgRNA sequences used in this study

sgRNA	Sequence
sg01	TCGTTTGCATTCTGTTTCTT
sg02	AGCACTTCTTCACCAAACCA
sg03	GTGTTTCTCTCGTTATCTGC
sg04	CCGTGCGATCGTGTTCAAAA
sg06	CACCGGGAAGCCATTCAGAG

**Table 3:** Plasmids used in this study

Strain	Description	Reference
pTLL108.1	*PacI::Pro-promoter::sgRNA*	(van Leeuwe et al. 2019)
pTLL109.2	*sgRNA::Terminator::PacI*	(van Leeuwe et al. 2019)
pFC332	Contains *cas9,* a hygromycin resistance cassette and a PacI site for introducing a sgRNA	(Nødvig et al. 2018; Seekles et al. 2021)
pCT001	*agsC* cut, 254 bp upstream of *agsC* CDS start codon	This study
pCT002	*agsC* cut, 252 bp downstream of *agsC* CDS stop codon	This study
pCT003	*agsE* cut, 232 bp upstream of *agsE* CDS start codon	This study
pCT004	*agsE* cut, 251 bp downstream of *agsE* CDS stop codon	This study
pJL006	*agsE* cut, 712 bp upstream of *agsE* CDS start codon	This study
pJL015	Tet-on promoter between 1000 bp flanks, the left flank 712–1712 bp upstream of the *agsE* start codon, while the right flank starts from the start codon	This study

**Table 4:** Primers used in this study

	Primer name	Primer Sequence
1	pTE1.Fwd	GTTTCCGCTGAGGGTTTAATACTCCGCCGAACGTACTG
2	pTE1.Rev	CTGTCTCGGCTGAGGTCTTAAAAAGCAAAAAAGGAAGGTACAAAAAAGC
3	agsC.gRnaUp.Fwd	AAGAAACAGAATGCAAACGAGTTTTAGAGCTAGAAATAGC
4	agsC.gRnaUp.Rev	TCGTTTGCATTCTGTTTCTTGACGAGCTTACTCGTTTCGT
5	agsC.gRnaDown.Fwd	TGGTTTGGTGAAGAAGTGCTGTTTTAGAGCTAGAAATAGC
6	agsC.gRnaDown.Rev	AGCACTTCTTCACCAAACCAGACGAGCTTACTCGTTTCGT
7	agsE.gRnaUp.Fwd	GCAGATAACGAGAGAAACACGTTTTAGAGCTAGAAATAGC
8	agsE.gRnaUp.Rev	GTGTTTCTCTCGTTATCTGCGACGAGCTTACTCGTTTCGT
9	agsE.gRnaDown.Fwd	TTTTGAACACGATCGCACGGGTTTTAGAGCTAGAAATAGC
10	agsE.gRnaDown.Rev	CCGTGCGATCGTGTTCAAAAGACGAGCTTACTCGTTTCGT
11	agsC Repair Oligo	CCATTCGCAACTGCAGACTTCAGCTGCCCAATTGTTCATCCTTCGTAGGGATAACAG
		GGTAATGGGGGACTTCTTCAC CAAACCATTCCCAGTAAGCACCAAGACCATACCAA
12	agsE Repair Oligo	AGATTTCACTCGTTAACGAAGCGTGCCTGTGTTTCTCTCGTTATCTAGGGATAACAG
		GGTAATGGGGGTGCGATCGTG TTCAAAAGGTTTCAATGTAGATGGTATATTGTCTA
13	agsE.Tet-on_cut.Fwd	CACCGGGAAGCCATTCAGAGGTTTTAGAGCTAGAAATAGC
14	agsE.Tet-on_cut.Rev	CTCTGAATGGCTTCCCGGTGGACGAGCTTACTCGTTTCGT
15	agsE.Uflank.Fwd	GAGTTTTTCAGCAAGATGACTACCGGACTACCACCAC
16	Tet-on:agsE.Uflank.Rev	TAAGACAGACCAGCCGAGGGTGAATGGCTTCCCGGTG
17	Tet-on.Fwd	CCCTCGGCTGGTCTGTCTTA
18	Tet-on.Rev	GGTGATGTCTGCTCAAGCG
19	Tet-on:agsE.Dflank.Fwd	CGCTTGAGCAGACATCACCATGAAGTGGGCTATTTCC
20	agsE.Dflank.Rev	TAGGAGATCTGCTCGAGAGGTAGTGGCGGATCAAACGC
21	pJET.Fwd	ACCTCTCGAGCAGATCTCCTAC
22	pJET.Rev	ATCTTGCTGAAAAACTCGAGCCATC
23	agsC.CK.Fwd	CCCACCCTAATCTATTCGGG
24	agsC.CK.Rev	TGTCGCTGTTATTCCAGTCA
25	agsE.CK.Fwd	AACAATTCCTATACGAGTGAGCTG
26	agsE.CK.Rev	AGACAGGAGTAGGATGATGAAGAG
27	Tet-on:agsE.CK.Fwd	TTTCCCACTTCATCGCAGCT
28	Tet-on:agsE.CK.Rev	TAGATGGAACCAAAGGTATT

To replace the *agsE* promoter for the inducible Tet-on cassette, sgRNA sg03 (cutting in its start codon; see above) was used combined with sgRNA sg06 (Table 2) that cuts 712 bp upstream in the promoter region (Fig. 1D). To this end, the latter sgRNA was introduced in plasmid pFC332 (as described above) resulting in pJL006 (Table 3). The sgRNA expression cassette was amplified in two parts using plasmids pTLL108.1 and pTLL109.2 (van Leeuwe et al. 2019)(Table 3) as templates for the 5’ (primer pair 1/14, Table 4) and 3’ (primer pair 2/13, Table 4) fragments that consist of the PacI::Pro-promoter::sgRNA and the sgRNA::Terminator::PacI, respectively. The overlapping amplicons were assembled by a Gibson reaction into a PacI-linearized and dephosphorylated pFC332 vector (Nødvig et al. 2018). Plasmid pJL015 (Table 3) containing the inducible *Tet-on:agsE* cassette was used as repair substrate. This vector was composed of the constitutively controlled Tet-on transactivator CDS followed by a synthetic promoter based on a septuple tetracycline operator and an *Aspergillus* minimal promoter amplified from pMT3 (Wanka et al. 2016) using primer pair 17/18 (Table 4). The inducible *Tet-on:agsE* cassette was preceded by 1000 bp upstream and downstream flanks of *agsE.* The upstream flank was obtained by amplifying the region upstream of the Cas9 cutting site in the promoter sequence with primer pair 15/16 (Table 4) and the downstream flank starting in the *agsE* start codon using primer pair 19/20 (Table 4). The Tet-on promoter and the 2 homologous 1000 bp flanks were assembled into the pJET backbone (Fig. 1D), which was amplified by using primer pair 21/22 (Table 4) and the commercial pJET template from CloneJET PCR Cloning Kit (ThermoFisher, Massachusetts, US) (Table 2). Primer pair 23/24 was designed to give a 9265 bp fragment in the wild-type and a 1098 bp band in the Δ*agsC* strain, while primer pair 25/26 was designed to give a 9075 bp fragment in the wild-type and a 1082 bp band in the Δ*agsE* strain. Primer pair 27/28 was designed to give a 1136 bp fragment in *Tet-on:agsE* strain while no band should be obtained in the wild type (forward primer binds to the Tet-on promoter).

### Transformation of A. niger

Protoplasts were prepared according to de Bekker et al. (2009) and transformation was done as described (Meyer et al. 2010). Transforming DNA was taken up in 20 μL STC (50 mM CaCl_2_, 10 mM Tris/HCl, 1.33 M sorbitol, pH 7.5) and added to 100 μL of the same buffer containing 10^6^ protoplasts. To obtain strain Δ*agsC*, protoplasts of MA234.1 were co-transformed with 1 μg pCT001, 1 μg pCT002 and 10 μL repair oligo 11 (Biolegio, Nijmegen, Netherlands; Table 4). Similarly, 1 μg pCT003, 1 μg pCT004 and 10 μL repair oligo 12 (Biolegio, Nijmegen, Netherlands; Table 4) were used to co-transform MA234.1 to obtain Δ*agsE*. To obtain strain MA234.1 *Tet-on:agsE*, protoplasts were transformed with 1 μg pCT003, 1 μg pJL006 and 2 μg pJL015 as donor DNA. 50 μL 60% PEG4000 was added and mixed gently before adding 1 ml 60% PEG4000 followed by incubation at room temperature for 5 min. This was followed by adding 2 ml STC, mixing the transformation mixture with 30 ml molten (50 °C) MM-ST (0.95 M sucrose, 0.6% agar, pH 6), and spreading it onto two square plates containing MM-S (0.95 M sucrose, 1.2% agar, pH 6). Transformants were selected using 150 µg ml^−1^ hygromycin B both in the top and bottom layer. Single transformants were selected on PDA plates containing 150 µg ml^−1^ hygromycin B followed by two purifications on PDA without antibiotic to get rid of the sgRNA plasmids.

### DNA isolation

*Escherichia coli* strain NEB® 10-beta was grown at 37 °C and 200 rpm in LB medium. *E.coli* plasmid DNA was isolated with the NucleoBond PC 100 Plasmid Miniprep Kit (Macherey Nagel, Düren, Germany), while genomic DNA of *A. niger* was isolated as described (Liu et al. 2000) with modifications. Mycelium was grown overnight at 30 ℃ and 200 rpm and harvested by using a 40 μm cell strainer (Corning, 352,340, New York, United States). After removing excess of water using filter paper, the mycelium was frozen in liquid nitrogen and homogenized with 2 steel beads at 25 Hz for 1 min in a Tissue lyzer II (Qiagen, Hilden, Germany). A total of 500 μL lysis buffer was added to 5–20 mg mycelium powder and incubated at room temperature for 10 min. This was followed by mixing with 150 μl 3 M potassium acetate buffer (pH 4.8) and centrifugation at 10,000 g for 1 min. The supernatant was centrifuged again at 10,000 g for 2 min after mixing with an equal volume of isopropanol. The pellet was washed with 70% ethanol and dissolved in 50 μL TE Buffer (NucleoBond PC 100 Plasmid Miniprep Kit, Macherey Nagel).

### Analysing micro-colony diameter and biomass

Bright field images of micro-colonies were converted to binary images by thresholding. The particle analysis tool in ImageJ was used to segment the micro-colonies using a size > 50 square pixel (i.e. > 100 µm^2^), to get rid of small debris, and a circularity between 0 and 1. The diameter of micro-colonies was calculated using the formula $2\cdot \sqrt{\frac{area}{\pi }}$ assuming that micro-colonies were spherical. Analysis was done using biological triplicates with a total of > 100 pellets. Data was analyzed using Levene’s Test for equality of variance detection and one-way ANOVA followed by Games-Howell/turkey post-hoc tests test for multiple comparisons (*p* ≤ 0.05). To determine biomass, mycelium of biological triplicates was filtered over filter paper, washed twice with distilled water, dried at 60 °C, and weighed. Biomass was analyzed using one-way ANOVA followed by Games-Howell post-hoc test for multiple comparisons (*p* ≤ 0.05).

### SDS-PAGE

Protein contained in 400 µL culture medium was precipitated overnight at −20 °C with four volumes pre-cooled acetone, pelleted at 4 °C at 20,000 g for 30 min, and dissolved in 20 µL loading buffer (20% glycerol, 4% SDS, 100 mM Tris–HCL pH 6.8, 0.01% bromophenol blue). Proteins were separated in 12.5% SDS-poly acrylamide gels using TGS buffer (30 g Tris base, 144 g glycine, and 10 g SDS L^−1^). Gels were stained in 0.02% CBB G-250, 5% Al_2_(SO_4_)_3_(14–18)-hydrate, 10% ethanol and 2% phosphoric acid and destained in 10% ethanol and 2% phosphoric acid (Krijgsheld et al. 2012).

### Massspectrometry:RP-nanoLC-MS/MS

Pellets were harvested and excess water was removed with tissue paper. A total of 50 mg wet weight mycelium was taken up in 100 μL lysis buffer (1 pill cOmplete protease inhibitor, [Roche, Switzerland] dissolved in 10 ml100 mM Tris, 10% SDS, pH 8.47) and centrifuged at 20,000 g for 30 min. 20 μL supernatant was loaded on a SDS PAGE gel (see previous section), ran for 2–3 cm, and stained with colloidal coomassie dye G-250 (Thermo Fisher Scentific, cat# 24,590). Similarly, proteins in the culture medium were separated by SDS PAGE and stained. Gel pieces containing the cellular or extracellular proteins were reduced, alkylated and digested overnight with trypsin at 37 °C. Peptides were extracted with 100% acetonitrile (ACN) and dried in a vacuum concentrator. Samples were resuspended in 10% (v/v) formic acid for UHPLC-MS/MS analysis.

Resuspended peptides were subjected to LC-LC MS/MS using a Thermo Ultimate 3000 coupled to an Orbitrap Exploris 480 mass spectrometer (Thermo Scientific, Bremen, Germany). To this end, peptides were loaded on a C18 PepMAP column (5 μm, 5 mm × 300 μm; Thermo Scientific, cat#160,454) using solvent A (0.1% formic acid) before being separated on an analytical column (Agilent Poroshell EC-C18, 2.7 μm, 50 cm × 75 μm). The extracellular proteins were eluted during a 46 min run using the following gradient: 9 − 13% solvent B (0.1 formic acid in 80% ACN) in 1 min, 13 − 44% solvent B in 37 min, 44–99% solvent B in 3 min, 99% solvent B for 4 min and back to 9% solvent B in 1 min. Similarly, the cellular proteins were eluted using a 104 min run with the following gradient: 9 -13% solvent B in 1 min, 13—44% solvent B in 95 min, 44—99% solvent B in 3 min, 99% solvent B for 4 min and back to 9% solvent B in 1 min. The mass spectrometer was operated in data-dependent mode. Full-scan MS spectra from m/z 375—1600 were acquired at a resolution of 60 000 at m/z 200 after accumulation to the standard target value. Cycle time of 1 s for the 46 min and 2 s for the 104 min gradient with standard AGC targets, respectively. HCD fragmentation was performed at normalized collision energy of 28.

### Proteomics data analysis

Raw data was analyzed with MaxQuant software (version 1.6.8.0) using label-free quantification (Cox et al. 2011). A false discovery rate (FDR) of 0.01 for proteins and peptides and a minimum peptide length of 7 amino acids were required. MS/MS spectra were searched against the database using the Andromeda search engine. Trypsin allowing N-terminal cleavage to proline was selected for enzyme specificity. Cysteine carbamidomethylation was selected as fixed modification, while protein N-terminal acetylation and methionine oxidation were selected as variable modifications. Up to two missing cleavages were allowed. Initial mass deviation of precursor ion was up to 7 ppm, mass deviation for fragment ions was 0.05 Da. Protein identification required one unique peptide to the protein group and match between run was enabled. In all cases, 4 biological replicates and at least 2 technical replicates were used. All correction analyses were carried out with Perseus software Version 1.6.10.0. All biological replicates had an R^2^ > 0.97. Proteins were considered present when they had been identified in at least 3 out of 4 biological replicates. Pfam and GO over-representation and signal peptide analysis were done as described (El-Gebali et al. 2019; Petersen et al. 2011).

### Cellulase activity assay

Cellulase activity was quantified using the filter paper activity assay (FPase) (Xiao et al. 2004). To this end, 7-mm diameter round Whatman No. 1 filter paper was incubated with 60 µL culture medium for 24 h at 50 °C. This was followed by a 5 min incubation at 95 °C after adding 120 μL DNS (10 g L^−1^ 3,5-dinitrosalicylic acid, 400 g L^−1^ KNa-tartrate and 16 g NaOH L^−1^). Aliquots of the samples (100 µL) were transferred to the wells of a flat-bottom plate and the A_540_ was determined using a Synergy HTX Microplate Reader (BioTek, Winooski, VT, USA). Calibration curves were made using different concentrations of glucose. Units of cellulase activity were defined as 1 µmol glucose or xylose being released in 1 min, respectively. Cellulase activity within the culture media of MA234.1 and *ΔagsE* cultures and mixtures thereof were analysed by one-way ANOVA, followed by either a Bonferroni or Dunnett's T3 test for multiple comparisons.

### Pathway analysis

Protein expression were calculated by using R package “DESeq2” and KEGG enrichment analyses was performed using the “phyper” function from the R package stats (Kanehisa and Goto 2000). The Benjamini–Hochberg was used to correct for significance p-value of 0.05.

## Results

### Impact of inactivation of agsC and agsE on morphology and biomass of A. niger and the pH of its culture medium

**Fig. 2:**
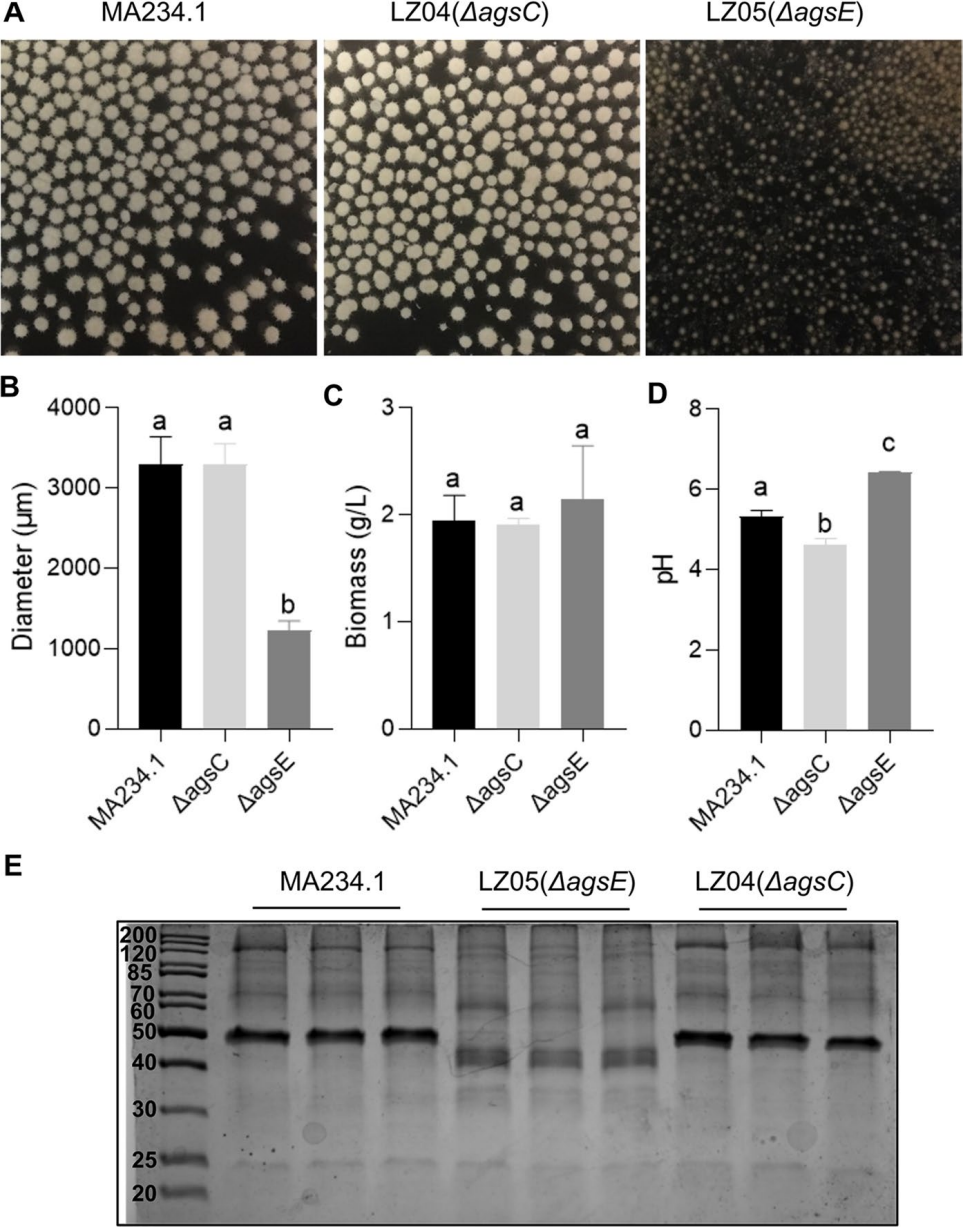
Morphology (**A**), diameter (**B**), biomass (**C**), pH (**D**) and SDS PAGE of proteins in the culture medium (**E**) of strains MA234.1, ΔagsC and ΔagsE. Different letters indicate statistical differences (**B**-**D**) as determined by a one-way ANOVA combined with a Games-Howell post-hoc test. Error bars indicate standard deviation

Genes *agsC* (ATCC64974_40950, AspGD) and *agsE* (ATCC64974_10510, AspGD) were inactivated in strain MA234.1 to study the relationship between α-(1,3)-glucan synthesis and biomass, micro-colony morphology, pH of the culture medium and the secretome. Strains LZ04 (Δ*agsC*) and LZ05 (Δ*agsE*) and their parental strain MA234.1 were pre-cultured in transformation medium with 25 mM xylose (TM-X) for 16 h and transferred to minimal medium with 25 mM xylose (MM-X) for a total culturing time of 48 h. Biomass of wild-type strain MA234.1 and the Δ*agsC* and Δ*agsE* strains were similar with 1.94 ± 0.24 g l^−1^, 1.91 ± 0.6 g l^−1^, and 2.15 ± 0.5 g l^−1^, respectively (Fig. 2C). Diameter of micro-colonies of MA234.1 (3304 ± 338 µm) and Δ*agsC* (3302 ± 252 µm) was also similar (Fig. 2AB). In contrast, Δ*agsE* developed smaller pellets with a diameter of 1229 ± 113 µm. Light microscopy revealed that Δ*agsE* forms such small micro-colonies due to reduced aggregation of both dormant and germinating spores (Supplemental Fig. 1). The pH of the culture media of MA234.1 (pH 5.2), Δ*agsC* (pH 4.6) and ΔagsE (pH 6.4) was different (Fig. 2D), implying that inactivation of α-(1,3)-glucan synthases impacts the secretion of organic acids. In addition, SDS PAGE indicated that the secretomes of Δ*agsC* and MA234.1 were similar but that the secretome of Δ*agsE* was different (Fig. 2E). Taken together, α-(1,3)-glucan synthase gene *agsE* is involved in colony morphology, in secretion of organic acids and in protein secretion, while *agsC* only affects pH of the culture medium.

**Fig. 3:**
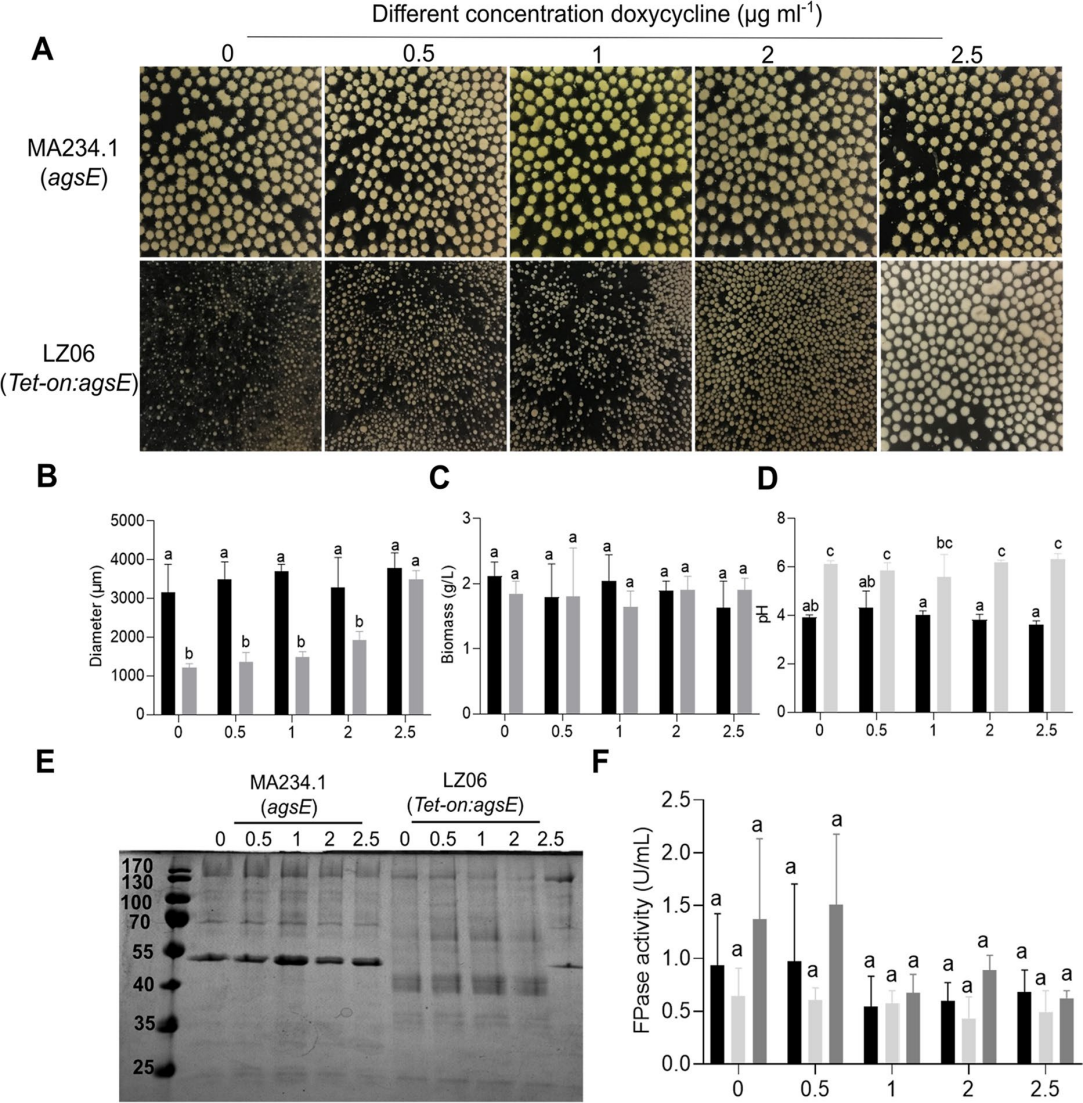
Morphology (**A**), diameter (**B**), biomass (**C**), pH (**D**), SDS PAGE of the proteins in the culture medium (**E**) and cellulase activity (**F**) of strains MA234.1 and LZ06 (*Tet-on:agsE*) at different concentrations of doxycycline in the culture medium. Dark and light shaded bars in B-F indicate strains MA234.1 and LZ06, respectively, while intermediate grey shaded bars in F represent a mixture of the culture media of both strains. Letters indicate statistical significance (**B**-**D**,**F**) as determined by a one-way ANOVA combined with a Turkey post-hoc test (**B**-**D**) and multiple t-test (**E**,**F**). Error bars indicate standard deviation

We aimed to complement the Δ*agsE* strain to confirm that its phenotypes are caused by the inactivation of the α-(1,3)-glucan synthase gene. However, the size of this gene (7533 bp) hampered complementation and therefore we used a different strategy by replacing the *agsE* promoter in the wild-type strain for the Tet-on promoter that is induced by doxycycline. This strategy enables to monitor the deletion phenotype (no doxycycline in the medium), the normal phenotype (x amount of doxycycline in the medium), as well as the over-expression phenotype (x + y amount of doxycycline in the medium). Strain LZ06 (*Tet-on:agsE*) was cultured in the presence of different concentrations of doxycycline both during the 16 h pre-culturing in TM-X and the 32 h culturing in MM-X. Morphology of the parental strain MA234.1 was not affected by the presence of 2.5 µg ml^−1^ doxycycline (3156 ± 725 µm versus 3784 ± 397 µm in absence and presence of the inducer, respectively). In contrast, micro-colony size of the *Tet-on:agsE* strain ranged from 1206 ± 113 µm (no doxyxcycline) to 1923 ± 224 µm (2 µg ml^−1^ doxycycline), to 3500 ± 224 µm (2.5 µg l^−1^ doxycycline) (Fig. 3A,B). Biomass of MA234.1 and *Tet-on:agsE* cultures were not statistically different, while pH was significantly higher in the *Tet-on:agsE* cultures compared to MA234.1 in the absence or presence of the inducer (Fig. 3C,D). Next, the secretome was evaluated by SDS-PAGE. Presence of doxycycline did not affect the protein profile of the wild-type (Fig. 3E). Notably, the protein profile of strain LZ06 was very similar to the wild-type in the presence of 2.5 µg l^−1^ doxycycline, while it was highly different at ≤ 2 µg l^−1^ inducer. These results show a relation between morphology and the protein profiles in the culture medium.

**Fig. 4:**
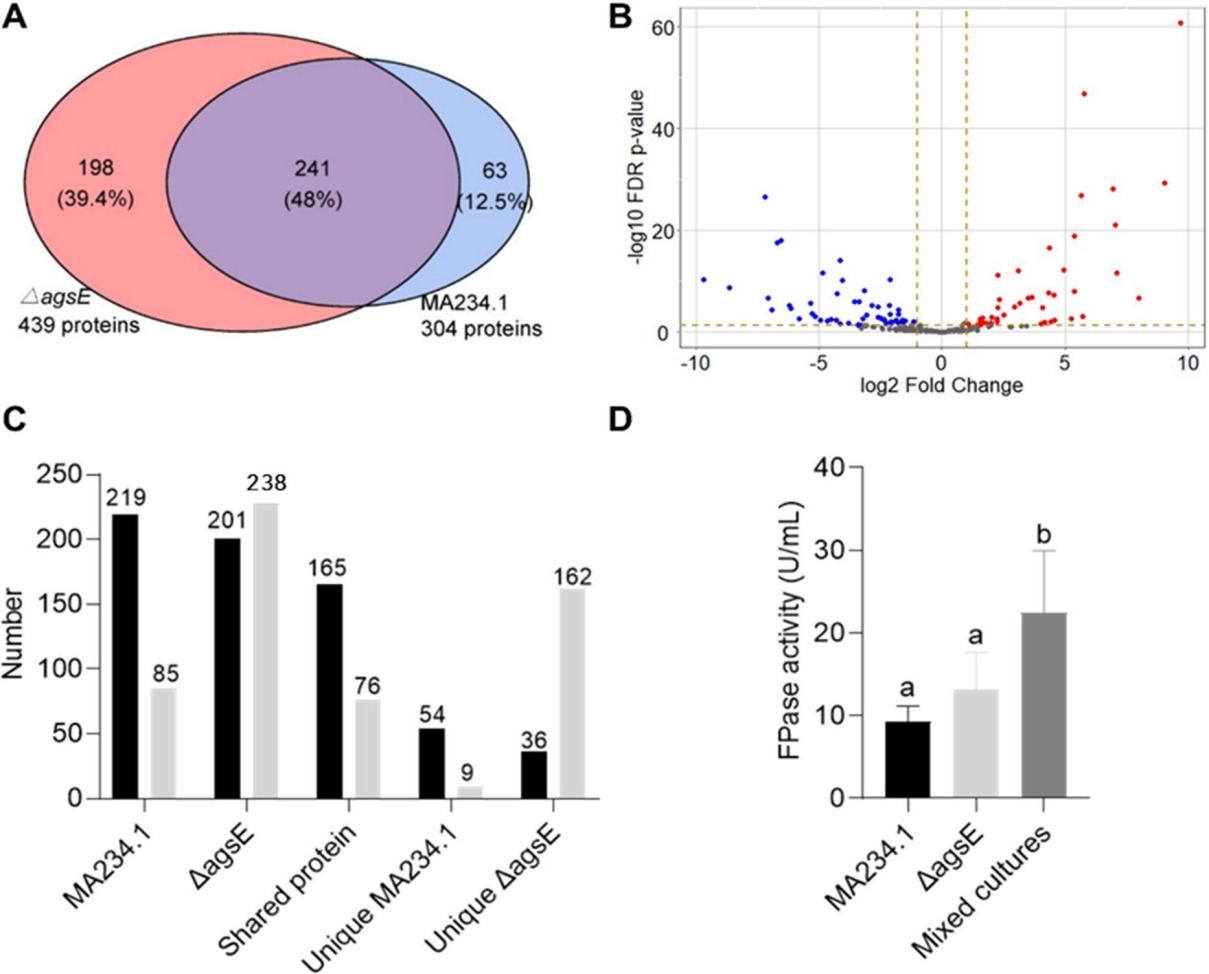
Total number of proteins (**A**) and up- and down-regulated proteins (**B**) in the culture medium of strains MA234.1 and Δ*agsE*. Red and blue dots in panel B indicate proteins that were ≥ twofold down- or up-regulated in Δ*agsE* cultures when compared to MA234.1 cultures, respectively. **A** and **B** comprise proteins with and without signal sequence, while (**C**) distinguishes between proteins with (black shaded bars) and without (light shaded bars) a signal sequence. **D** Cellulase activity from liquid shaken cultures of MA234.1 and Δ*agsE*. Proteins were analyzed after a total culturing time of 48 h. Statistical differences was determined by a one-way ANOVA combined with a Bonferroni or Dunnett's T3 test for multiple comparisons. Letters indicate statistical differences, while error bars indicate standard deviation

Cellulase activity of cultures MA234.1 and the *Tet-on:agsE* strain was evaluated in the absence and presence of doxycycline and in the mixture of the culture media of the two strains grown in the same concentration of the inducer. No statistical differences were observed in cellulase activity between the strains at the different concentrations of the inducer and also between the pure and mixed culture media. Yet, a trend towards higher cellulase activity was observed in the absence of inducer or in the presence of 0.5 µg l^−1^ inducer, suggesting that large wild-type and small LZ06 micro-colonies show synergistic cellulase activity (Fig. 3F). Similarly, cellulase activity of the Δ*agsE* cultures was 41.6% higher than that of the wild type cultures (Fig. 4D) and the 1:1 mixed culture media of MA234.1 and Δ*agsE* exhibited 2.4- and 1.7-fold higher activity compared to the cultures of MA234.1 and Δ*agsE*, respectively. Taken together, *agsE* influences the pH, micro-colony morphology and cellulase of *A.niger* and the protein profiles in the culture medium.

### Secretome of MA234.1 and ΔagsE

Secretome analysis was performed of MA234.1 and Δ*agsE* revealing a total number of 304 and 439 proteins, respectively, of which 219 and 201 are predicted to have a signal peptide for secretion (Fig. 4C; Supplemental Tables 1 and 2). Thus, strain Δ*agsE* releases more proteins without signal peptides when compared to MA234.1. A total of 241 proteins was identified in the culture media of both MA234.1 and Δ*agsE*, while 63 and 198 proteins were unique for these strains, respectively (Fig. 4C; Supplemental Tables 3-5). Within the set of 241 shared proteins, 165 and 76 were predicted to have or lack a signal peptide for secretion (Fig. 4C; Supplemental Table 3), respectively, while 26 and 66 proteins within the set of 241 shared proteins were significantly > twofold down- and up-regulated in Δ*agsE* when compared to MA234.1 (Fig. 4B; Supplemental Table 6). Of the 63 unique proteins of the wild-type strain, 54 had a predicted signal sequence for secretion (Fig. 4C; Supplemental Table 4). In contrast, only 36 proteins contain a signal sequence for secretion within the set of 198 unique proteins released by strain Δ*agsE* (Fig. 4C; Supplemental Table 5). Together, these data show that the Δ*agsE* strain releases less proteins with a signal sequence when compared to the wild-type and that the set of unique proteins with a signal sequence is also smaller.

**Fig. 5:**
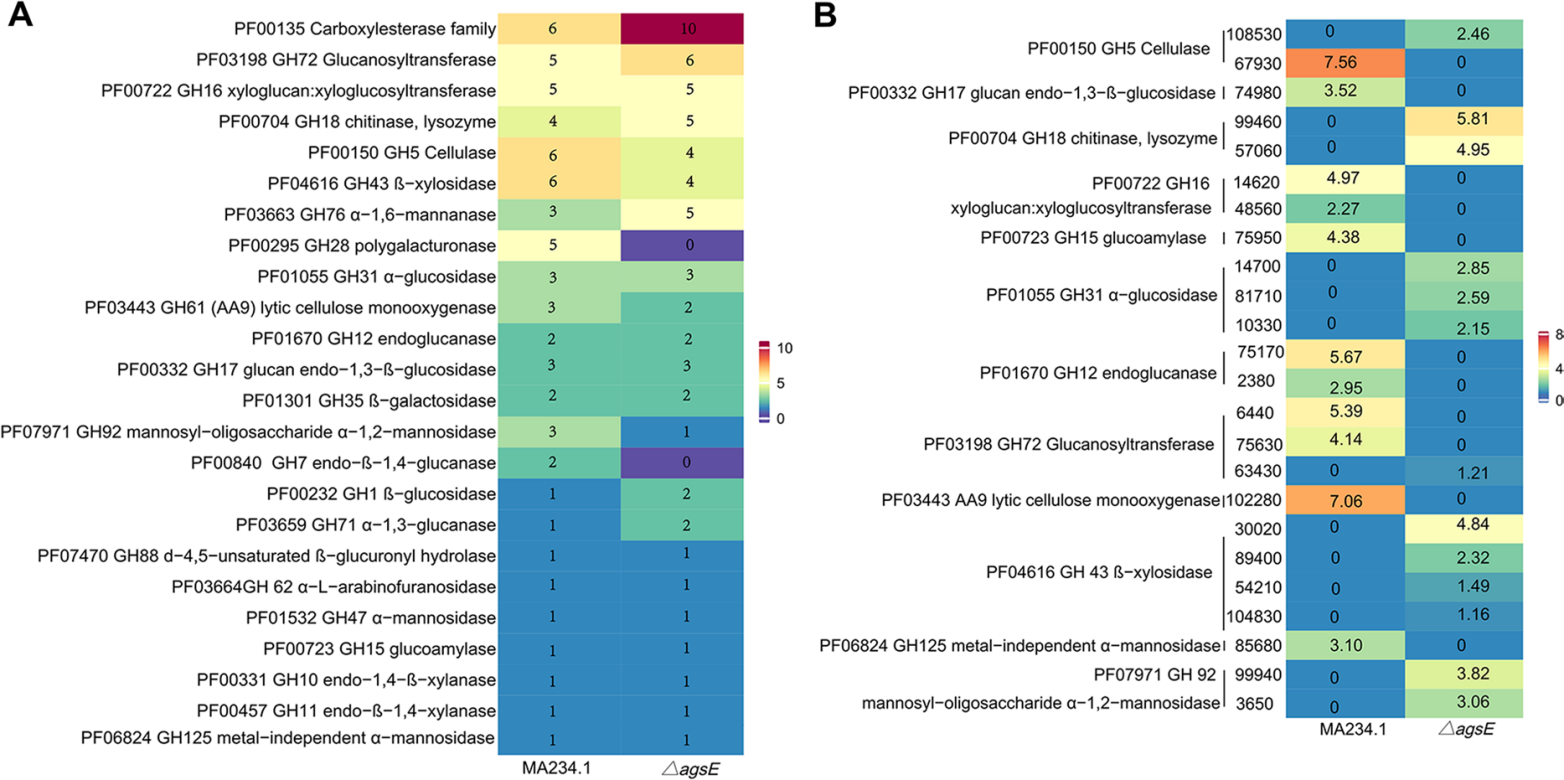
Protein family (Pfam) analysis of Cazyme proteins in the secretomes of strains MA234.1 and LZ05 (*ΔagsE*) (**A**) and the significantly up- or down-regulated Cazymes of LZ05 (*ΔagsE*) compared to MA234.1 (proteins present in cultures of both MA234.1 and *ΔagsE*) (**B**). The number in (**A**) is the number of proteins identified in the cultures; the number in (**B**) represent the log fold change (log2^(x)^ = y, x represent expression fold change, y is the number shown in panel B. 0 in blue is when x = 1; set as control). The number in B annotation represents X in the proteinID ATCC64974_X of N402

The 219 proteins with a signal peptide identified in the secretome of MA234.1 belong to 103 protein families (Supplemental Table 7). This set included 6 carboxylesterases (PF00135), 5 GH5 cellulases (PF00150), 5 GH28 polygalacturonases (PF00295), 5 GH16 glucanases / galactanases (PF00722) and 5 GH43 hemicellulases (PF04616) (Fig. 5A). The 85 proteins that are released into the culture medium of MA234.1 that do not have a predicted signal peptide belong to 67 protein families (Supplemental Table 7). Eukaryotic aspartyl protease (PF00026) and pectate lyase superfamily protein (PF12708) were represented by 2 proteins each. The 201 proteins with a signal peptide for secretion that were released by strain *ΔagsE* represent 100 protein families (Supplemental Table 8). This included 10 carboxylesterases (PF00135), 5 GH16 glucanases / galactanases (PF00722), 4 GH76 α-mannanases (PF03663), 3 α-amylases (PF00128) and 3 GH5 cellulases (PF00150) (Fig. 5A). The 228 proteins without a predicted signal sequence that were identified in the culture medium of Δ*agsE* belonged to 179 protein families (Supplemental Table 8). PF00083 sugar (and other) transporter (7 proteins), PF00248 aldo/keto reductase family (4 proteins), PF00326 prolyl oligopeptidase family (3 proteins) and PF01557 fumarylacetoacetate (FAA) hydrolase family (3 proteins) were the families with most representatives.

The set of 165 proteins with a predicted signal peptide that are shared between wild-type strain MA234.1 and strain *ΔagsE* represents 88 protein families with PF00722 GH16 glucanases / galactanases (5 proteins), PF00150 GH5 cellulase (3 proteins), PF00704 GH18 chitinases / endo-β-N-acetylglucosaminidases / carbohydrate binding modules / xylanase inhibitors (3 proteins), PF01055 GH31 α-glucosidases / α-xylosidases / isomaltosyltransferases / glucoamylases (3 proteins), and PF04616 GH43 hemicellulases (3 proteins) having most representatives (Supplemental Table 9). The shared set of 76 proteins without a signal peptide belonged to 64 protein families with PF01042 endoribonuclease L-PSP and PF12708 pectate lyase superfamily being most represented with 2 proteins each (Supplemental Table 9). The 54 unique proteins with a predicted signal peptide in the culture medium of MA234.1 belonged to 31 protein families including 5 GH28 polygalacturonases (PF00295), 2 GH5 cellulases (PF00150), 2 GH7 cellulases/β-1,4-glucanases (PF00840) and 2 pectinesterases (PF01095) (Supplemental Table 10). No GH family was identified when we analzed the 9 unique proteins without a signal peptide in the secretome of MA234.1 (Supplemental Table 10). The 36 unique proteins of Δ*agsE* with a predicted signal peptide were classified into 24 protein families. The PF00135 carboxylesterase family (4 proteins), and the PF03663 GH76 α-mannanases (2 proteins) were among the most prominent protein families (Supplemental Table 11). Strain Δ*agsE* secreted 162 unique proteins without predicted signal peptides that belong to 129 protein families (Supplemental Table 11). PF00083 sugar (and other) transporters (7 proteins) was the most represented family. MA234.1 and Δ*agsE* secreted members of 3 (GH28, GH5, GH7) and 3 (GH76, GH1, GH18) unique GH families, respectively, suggesting they have complementary activity on plant substrates.

A set of 57 proteins was significantly > twofold up-regulated in Δ*agsE* compared to MA234.1. This set included 4 GH43 hemicellulases (PF04616), 3 GH31 α-glucosidases / α-xylosidases / isomaltosyltransferases / glucoamylases (PF01055), 2 GH18 chitinases / endo-β-N-acetylglucosaminidases / carbohydrate binding modules / xylanase inhibitors (PF00704), 2 GH92 PF07971 α-mannosidases, 1 GH 72 PF03198 glucanosyltransferase and 1 PF00150 cellulase (Fig. 5B, Supplemental Table 12). In contrast, 46 shared proteins were > twofold down-regulated in Δ*agsE*, including 2 GH16 glucanase / galactanase (PF00722), 2 GH12 β-glucanases (PF01670), 1 GH17 β-glucan endohydrolases (PF00332), 1 GH15 α-glucosidase (PF00723), 2 GH 72 glucanosyltransferase (PF03198), 1 AA9 lytic cellulose monooxygenase( PF03443), 1 GH 125 exo-α-1,6-mannosidase (PF06824) and 1 cellulase (PF00150) (Fig. 5B, Supplemental Table 12). Together, these data support that MA234.1 and LZ05 may have complementary activity on cellulose and plant substrates.

## Discussion

Deletion of α-1–3 glucan synthase genes impacts aggregation of germinating spores and, as a consequence, results in smaller *A. nidulans* micro-colonies in liquid cultures (He et al. 2014; Yoshimi et al. 2013). Here it is shown that inactivation of the α-1–3 glucan synthase gene *agsE*, but not *agsC,* of *A. niger* also results in smaller micro-colonies. These small micro-colonies not only result from reduced aggregation of germinating spores but also of reduced clumping of dormant spores. Formation of small micro-colonies by the Δ*agsE* strain was associated with changes in extracellular pH and protein profiles in the culture medium. In fact, data suggest that the wild-type strain and the Δ*agsE* strain have complementary activity on plant substrates.

Recently, evidence has been provided that α-1–3 glucan plays an important role as a structural polysaccharide in the cell wall of filamentous fungi (Kang et al. 2018; Ehren et al. 2020). Therefore, inactivation of α-1–3 glucan synthase genes may impact cell wall integrity and therefore growth of the fungus. Yet, inactivation of *agsC* and *agsE* did not impact biomass formation in cultures of *A. niger.* Apparently, compensatory mechanisms such as those mediated by the *crh* gene family are available to provide a functional cell wall. Crh proteins are chitin transglycosylases transferring soluble chitin molecules to chitin (homotransglycosylation) or β-1,3 and β-1,6-glucan (heterotransglycosylation) acceptors (Mazáň et al. 2013). As such, they are involved in cross-linking chitin and β-glucan in the cell wall. Deletion of the seven *crh* genes in a strain of *A. niger* that is deficient in α-1,3-glucan results in a growth defect and an increased sensitivity towards a cell wall perturbing agent (van Leeuwe et al. 2020), which is not observed in the wild-type. Four of these seven proteins (ATCC64974_88180; ATCC64974_48560; and ATCC64974_14620; ATCC64974_70810) were found both in wt and Δ*agsE*. Of these, ATCC64974_14620 was down regulated in the deletion strain. Apart from the Crh proteins also β-1,3-glucanosyltransferases can play a role in cell wall intregrity. These proteins are involved in the formation of branched glucans in the cell wall in fungi and yeasts and also function in cell wall assembly and rearrangement (Mouyna et al., 2000a, 2000b). A total of 5 and 6 predicted β-1,3-glucanosyltransferases are found in the secretome of the reference strain and Δ*agsE,* respectively. The β-1,3-glucanosyltransferase ATCC64974_55200 was unique in the secretome of the deletion strain, while ATCC64974_63430 was up-regulated. On the other hand, ATCC64974_6440 and ATCC64974_75630 were down-regulated. Together, the secretome data suggest that cell wall assembly and re-arrangement is different in the reference strain and strain Δ*agsE*.

The pH of the culture medium changed from 5.2 in the case of the wild-type to 4.6 and 6.4 for Δ*agsC* and Δ*agsE,* respectively. It is not clear why the deletion strains have a different effect on pH but they may differ in secretion of organic acids. The *agsC* gene had no apparent impact on the secretome. This suggests that the lower pH in the culture medium of the Δ*agsC* strain has no effect on protein secretion. Inactivation of *agsE* did impact protein release into the medium. Secretome analysis showed a total number of 304 and 439 proteins that had been released into the culture medium of MA234.1 and strain Δ*agsE*, respectively, of which 219 and 201 have a signal peptide for secretion. Thus, Δ*agsE* releases more proteins without signal peptides when compared to MA234.1. It is not yet clear why the deletion strain releases more proteins that lack a signal sequence. Possibly, part of these proteins are released via non-classical secretion including autophagy- and extracellular-vesicle-dependent pathways. Notably, HSP70 that was found in the Δ*agsE* secretome but not in that of the reference strain has been detected in extracellular vesicles of several fungi (Miura and Ueda 2018). Also, pyruvate kinase and L3 ribosomal protein that were found in the Δ*agsE* secretome but not in that of the reference strain have been proposed to be secreted via unconventional secretion in *Saccharomyces cerevisae* and *Aspergillus fumigatus,* respectively. Proteins may also be released due to hyphal damage or lysis. A wide variety of cytoplasmic and plasma membrane proteins are released in the medium of Δ*agsE* including a variety of ribosomal proteins, sugar and amino acid transporters and proteins involded in central metabolism. Smaller micro-colonies may be more sensistive to mechanical damage due to shear stress in liquid shaken cultures. This would also explain why small wild-type colonies show more release of proteins without signal sequence in the culture medium when compared to large wild-type micro-colonies (Lyu et al. 2023). Moreover, reduced presence or even absence of α-1–3 glucan may impact the cell wall integrity and thereby induces hyphal lysis. Yet, biomass of the cultures was shown not to be affected and there is evidence of mechanisms to compensate for the absence of α-1–3 glucan in *A. niger* (see above).

A total of 219 and 201 proteins with a signal peptide for secretion were identified in the culture media of MA234.1 and the Δ*agsE* strain, of which 165 were shared between the two strains. Of the total number of proteins with a signal sequence for secretion, 5 have never been reported to be part of the secretome before (Supplemental Table 13). Part of the proteins that were found in the secretomes of both MA234.1 and Δ*agsE* were differentially expressed when compared to the other strain. The 26 proteins that were down-regulated in Δ*agsE* when compared to the reference strain included GH16 glucanases / galactanases, GH12 β-glucanases, a GH17 β-glucan endohydrolases and a GH15 α-glucosidase (Fig. 5). On the other hand, the 66 proteins that were up-regulated in Δ*agsE* included GH43 hemicellulases, GH31 α-glucosidases / α-xylosidases / isomaltosyltransferases / glucoamylases, GH18 chitinases / endo-β-N-acetylglucosaminidases / carbohydrate binding modules / xylanase inhibitors and GH92 α-mannosidases. The 54 unique proteins with a predicted signal peptide in the culture medium of MA234.1 included GH28 polygalacturonases, GH5 cellulases, GH7 cellulases/β-1,4-glucanases and 2 pectinesterases. On the other hand, the 36 unique proteins with a predicted signal peptide of strain Δ*agsE* included carboxylesterases, GH76 α-mannanases, GH1 β-glucosidase, GH61 lytic polysaccharide monooxygenases and GH71 α-1,3-glucanase. Notably, strain Δ*agsE* did not release a unique GH5 cellulase when compared to MA234.1, implying we would not expect a synergistic cellulase activity as we found by mixing the culture media. The reason for this is not yet clear. Nevertheless, our data strongly suggest that the wild-type and the Δ*agsE* strains have complementary activity on plant substrates.

It has been reported that small micro-colonies release more protein than large micro-colonies (Tegelaar et al. 2020; Lyu et al. 2023). This may be explained by the fact that expression of genes of secreted proteins mainly occurs in a peripheral shell of the pellet and that, therefore, pellets with a radius ≤ of the width of the expression shell would be more productive in terms of protein secretion (Tegelaar et al. 2020). However, the results of this study do not support the finding that smaller micro-colonies secrete more protein in the culture medium. The increased amounts of protein as observed on SDS PAGE is mainly explained by release of proteins without a signal sequence. We propose that inactivation of *agsE* stimulates protein secretion due to formation of smaller micro-colonies formation but at the same time inhibits protein secretion due to for instance changes in cell wall composition, pH of the culture medium, cell lysis, or changes in activity of signalling pathways.

## Supplementary Information

Below is the link to the electronic supplementary material.

Supplementary file1 (DOCX 198 kb)

Supplementary file2 (XLSX 62 kb)

Supplementary file3 (XLSX 39 kb)
