# Supplementary material for: The α-(1,3)-glucan synthase gene agsE impacts the secretome of Aspergillus niger
Source: Antonie Van Leeuwenhoek. 2023 Jun 14;116(9):867–82. doi: 10.1007/s10482-023-01853-w (PMC10371888; doi:10.1007/s10482-023-01853-w)
Supplement: Supplementary file 1 — Supplementary file1 (DOCX 198 kb) [file 10482_2023_1853_MOESM1_ESM.docx]

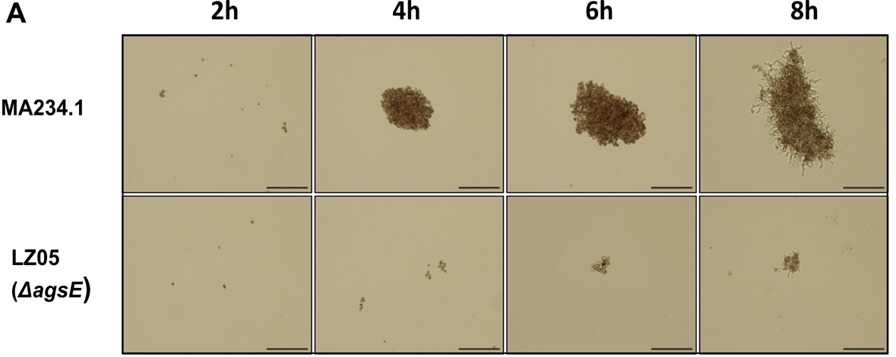


**Supplemental Figure 1.** Light microscopy of individual spores/germlings of MA234.1 and ΔagsE as well as their aggregates at different time points (Bar represents 100 µm)
